# Health Is a Duty

**Published:** 1870-07

**Authors:** 


### HALL'S

### JOURNAL OF HEALTH.

Vol. XVII. — JULY, 1870. — No. VII.

### HEALTH IS A DUTY.

It is just as self-evident a truth that

AVOIDABLE DISEASE IS A CRIME.

All callings in life have their duties and responsibilities; the first great duty, because it is an honesty, is to do our work in the best manner possible to the doer of the work, that the patron of that work may have the worth of his money. If the work is not done in the best manner, the payer for that work is to that extent cheated. There is no reputable calling in life which can be as well performed by a sick man, as by one who is in good health. Hence any man who is not in good health, and receives pay from another for what he does, is a perpetual cheat. The day laborer cannot do as much work if he is sick, nor can he do his work as well as if in good health.

Nor can the brain worker, the student, the clerk, the secretary, the foreman, the physician, the lawyer, the clergyman perform his duties as thoroughly if sick, as if well.

If the lawyer is sick, he cannot bring that concentration of mind upon a case, nor have that clearness of perception, necessary to bring the cause to the safest and speediest conclusion.

Of all the professions, the physician requires the clearest head, the most mature judgment, and the keenest discrimination. It is a common admission, that the physician who prescribes for himself in any grave case, is sure to hasten his dissolution.

And as to the clergyman, humanly speaking, a soul may be lost if his brain fails to act clearly, logically at the bedside of the dying, or in those critical seasons when the mind of an inquirer after the right way is balancing itself for eternity. Many a time does a man decide for or against, for the want of a proper word, or illustration, or sentiment, put in at a critical time.

The teacher, too, owes it as a duty to every pupil to be always in good health, so that his explanations shall be clear, made with earnestness, precision, and power. Millions of backs have been thrashed, millions of heads pummeled, millions of hearts crushed, millions of ambitions stunted by dyspeptic teachers.

And now coming into the sacred precincts of the family, many an industrious husband has been ruined by a wife forever complaining of some one or other of a million of ailments, arising nine times out of ten, from attempting to do too much, or from indiscretion in changing dress, or unnecessary exposures or hurry.

In a tenfold greater number of cases are the hearts and feelings of excellent wives wounded and crushed by the unappreciation of dyspeptic, gormandizing husbands, with their incessant growlings and complainings and fault-findings and thunder-gust looks; and how are children's hearts left bruised and bleeding every day from the impatience, and harshness, and despotism of parents whose tempers have been soured by disease of their own bringing on.

There are persons whose natural tempers are amiable, genial, and loving, but in a single hour, from some indiscretion in eating, the whole nature is changed, and all the evil passions run riot in their exhibitions; you never can tell in what mood you will find this class of persons, nor can you make any calculations for an hour ahead. A drunkard is sometimes as funny as a fool; at others so good-natured that it is impossible not to feel like excusing him; but the gormandizing dyspeptic, while under the influence of an attack, is neither generous nor gentle for one single half second of time; on the contrary, all the meaner traits of character shoot up their serpent heads, and there is spite, and impatience, and avarice, and uncontrollable ill nature.

Thus it is, that while health prepares for the proper performance of the duties of life and its amenities, sickness unfits for everything that is good; unfits for genial society and for the diffusion of social enjoyment, as would drunkenness or any other bestiality. Hence dyspepsia seems to be quite as much a crime as drunkenness, and is just as inexcusable because it is as avoidable; and if one avoidable disease is a crime against one's self, against the family relation, and against society, so is any other avoidable disease. Therefore health is a duty and disease a crime.

